# Identification of modules and novel prognostic biomarkers in liver cancer through integrated bioinformatics analysis

**DOI:** 10.1002/2211-5463.12983

**Published:** 2020-10-27

**Authors:** Bo Shen, Kun Li, Yuting Zhang

**Affiliations:** ^1^ Department of Hepatobiliary Surgery People's Hospital of Yichun City China; ^2^ Department of Liver Diseases People's Hospital of Yichun City China

**Keywords:** competitive endogenous RNAs, liver cancer, microRNA, prognosis, weighted gene coexpression network analysis

## Abstract

Liver cancer is a common malignant tumor with poor prognosis. Due to the lack of specific clinical manifestations at early stages, most patients are already at advanced stages of the disease by the time of diagnosis. Identification of novel biomarkers for liver cancer may thus enable earlier detection, improving outcome. MicroRNAs (miRNAs) are small endogenous noncoding RNAs of 18–22 nucleotides in length, which have a regulatory role in the expression of target proteins. Increased evidence suggests that miRNAs are abnormally expressed in a variety of cancer malignancies. Here, we combined RNA sequencing data and clinical information from The Cancer Genome Atlas Liver Hepatocellular Carcinoma database for weighted gene coexpression network analysis to identify potential miRNA prognostic biomarkers. We constructed nine coexpression modules, allowing us to identify that miR‐105‐5p, miR‐767‐5p, miR‐1266‐5p, miR‐4746‐5p, miR‐500a‐3p, miR‐1180‐3p and miR‐139‐5p are significantly associated with liver cancer prognosis. We found that these miRNAs exhibit significant association with prognosis of patients with liver cancer and confirmed the expression of these miRNAs in liver cancer tissues. Multivariate Cox regression analysis showed that miR‐105‐5p and miR‐139‐5p may be considered as independent factors. In summary, here we report that seven miRNAs have potential value as prognostic biomarkers of liver cancer.

## Graphical abstract

In this study, we constructed nine coexpression modules and identified that miR‐105‐5p, miR‐767‐5p, miR‐1266‐5p, miR‐4746‐5p, miR‐500a‐3p, miR‐1180‐3p and miR‐139‐5p were differentially expressed in liver cancer. These miRNAs exhibited a significant association with prognosis of patients with liver cancer. Among them, miR‐105‐5p and miR‐139‐5p may be considered as independent prognostic factors. Thus, seven miRNAs could be prognostic biomarkers for liver cancer.

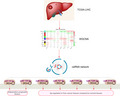

**Abbreviations**
AIC: Akaike information criterion
BP: biological processes
CC: cellular component
ceRNA: competitive endogenous RNA
FC: fold change
GO: Gene Ontology
KEGG: Kyoto Encyclopedia of Genes and Genomes
LIHC: Liver Hepatocellular Carcinoma
lncRNA: long noncoding RNA
ME: module eigengene
MF: molecular function
miRNA: microRNA
MM: Module Membership
RT‐qPCR: reverse‐transcription quantitative PCR
TCGA: The Cancer Genome Atlas
WGCNA: weighted gene coexpression network analysis

Liver cancer is one of the most common cancers worldwide and the second leading cause of cancer‐related deaths [1, 2]. Despite much progress in diagnosis and treatment, the prognosis of patients with liver cancer is still poor. Due to the lack of specific clinical manifestations in the early stage, most patients are already in advanced stages of symptoms and miss the opportunity to undergo radical resection. Therefore, identification of liver cancer pathogenesis contributes to early diagnosis, choice of treatment methods, determination of follow‐up timetable, and prognosis assessment, which can significantly prolong the survival time of patients with liver cancer [3].

Increased evidence suggests that microRNAs (miRNAs) are abnormally expressed in a variety of malignancies and are closely related to the pathogenesis of cancers, including liver cancer. miRNAs participate in the development of liver cancer as tumor suppressor genes or oncogenes. Therefore, further study of miRNA expression patterns and effects can provide new diagnostic or therapeutic targets for liver cancer. miRNAs are small endogenous noncoding RNAs, 18–22 nucleotides in length, which have a regulatory role in the expression of target proteins via inhibiting protein translation or enhancing down‐regulation of mRNA transcripts [4]. Long noncoding RNAs (lncRNAs) are a class of noncoding RNA transcripts; furthermore, their abnormal gene expressions promote tumor formation, progression, and metastasis, including liver cancer. In the cytoplasm, lncRNAs can regulate the expression of miRNA targets by competitively binding miRNAs to act as competitive endogenous RNA (ceRNAs) [5]. The ceRNA hypothesis shows that in the lncRNA–miRNA–mRNA ceRNA network, lncRNA competitively binds miRNA by sharing miRNA response elements and indirectly regulates mRNA expression levels [6]. At the same time, miRNAs negatively regulate gene expression at the posttranscriptional level by binding to sequences (mostly located in the 3′ UTR) and are partially complementary on their target mRNA [7].

Weighted gene coexpression network analysis (WGCNA) is one of the commonly used methods in coexpression module correlation analysis, which is widely applied in various biological processes (BP), especially for the identification of candidate biomarkers or therapeutic targets for many malignant tumors [8]. WGCNA is helpful to find associations between genes in different coexpression modules. Herein, a coexpression network was constructed through WGCNA to analyze the liver cancer expression profile dataset from The Cancer Genome Atlas (TCGA), which was used to explore possible carcinogenic mechanisms and potential hub genes as prognostic biomarkers. Then, we constructed a ceRNA regulatory network to understand the progress of liver cancer. Finally, based on the hub miRNAs, we constructed seven‐miRNA modules. The seven miRNAs were confirmed between 22 pairs of hepatocellular carcinoma tissues and adjacent normal tissues by reverse‐transcription quantitative PCR (RT‐qPCR).

## Materials and methods

### Data collection and preprocessing

The level 3 RNA sequencing data of Liver Hepatocellular Carcinoma (LIHC) were retrieved from TCGA data portal (https://cancergenome.nih.gov/), containing 371 liver cancer samples and 50 normal tissue samples. The mRNAs and lncRNAs were identified after annotation using Refseq transcript ID and Ensembl gene ID. A total of 335 liver cancer samples with complete clinical information in TCGA database were included in our study. The clinical information of patients with liver cancer included TNM, stage, grade, age and sex. The detail information of the dataset is shown in Table 1.

**Table 1:** The detail information of liver cancer samples from TCGA database. SD, standard deviation.

Characteristics	Overall (*N* = 335)
Sex, *n* (%)	
Male	231 (69.0%)
Female	104 (31.0%)
Age (years), *n* (%)	
Mean (SD)	59.2 (13.4)
Median [minimum, maximum]	61.0 [16.0, 90.0]
Status, *n* (%)	
Alive	221 (66.0%)
Dead	114 (34.0%)
Grade, *n* (%)	
G1	50 (14.9%)
G2	158 (47.2%)
G3	109 (32.5%)
Missing	18 (5.4%)
Stage, *n* (%)	
I/II	234 (69.9%)
III/IV	80 (23.9%)
Missing	21 (6.3%)
T, *n* (%)	
T1/T2	248 (74.0%)
T3/T4	84 (25.1%)
Missing	3 (0.9%)
N, *n* (%)	
N0	234 (69.9%)
N1	3 (0.9%)
Missing	98 (29.3%)
M, *n* (%)	
M0	243 (72.5%)
M1	3 (0.9%)
Missing	89 (26.6%)

**Fig. 1:**
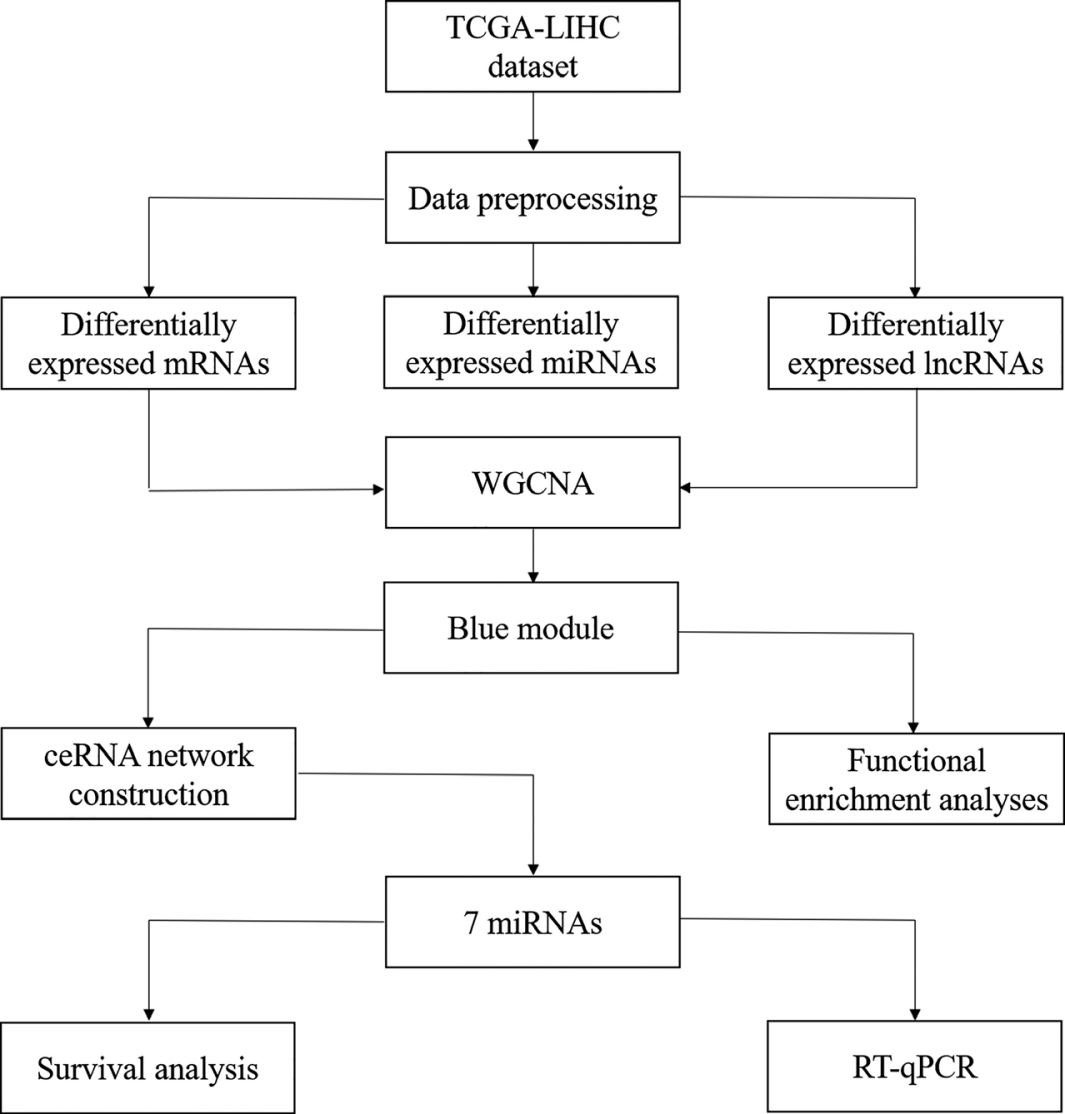
The overall workflow in our study.

We obtained the log_2_ (reads per million mapped reads (RPM) + 1) miRNA expression profile from TCGA database using the University of California Santa Cruz Xena (http://xena.ucsc.edu/). All data were downloaded in September 2017. The overall workflow of our study is shown in Fig. 1.

### Differential expression analysis

The raw data from TCGA–LIHC database were filtered and normalized using the edgeR package in r 3.6.0 [9]. The lncRNAs, miRNAs or mRNAs with adjusted *P* < 0.05 and |logFC| > 1 between 371 liver cancer samples and 50 normal tissue samples were identified to be differentially expressed via the edgeR package. The *P* value was adjusted using the Benjamini‐Hochberg (BH) method.

### WGCNA

The coexpression network was constructed through WGCNA package version 1.49 [10]. To build a coexpression module, we filtered the power values. As an important parameter, power value could mainly affect the scale independence and the mean connectivity degree of coexpression modules. Scale independence and average connectivity analysis of modules with different power values from 1 to 30 were performed by gradient test. When the scale independent value was equal to 0.85, the appropriate power value was confirmed. After constructing coexpression modules, the gene information in each module was extracted and cluster analysis was performed at the appropriate threshold value. The relationships among different coexpression modules were analyzed. The strength of the relationship was performed using heatmap package (strong or weak degree).

### Module–trait relationship analysis of liver cancer

Two methods were used to identify the module–trait relationships. Module–trait relationships were assessed by the correlation between the module eigengene (ME) and clinical traits. ME, as the major component for principal component analysis of genes in a module with the same expression pattern, may reflect the entire features of genes in a module. The clinical traits included TNM, stage, grade, age and sex. The correlation between ME and clinical traits was analyzed by Pearson's correlation tests, and *P* < 0.05 was considered to be significantly correlated. The module with the highest correlation coefficient and *P* < 0.05 was considered as a meaningful module.

In the intramodular analysis, for each expression profile, gene significance was calculated as the absolute value of the correlation between the gene expression profile and each clinical feature. Module Membership (MM) was defined as the correlation of expression profile and each ME. MM of a gene may be used to stand for the membership of the gene with respect to the module. Therefore, genes with a high significance for clinical traits and MM were identified.

### Identification of interested module and function enrichment analysis

The gene enrichment analysis of the genes in the meaningful module was performed including Kyoto Encyclopedia of Genes and Genomes (KEGG) pathway and Gene Ontology (GO) through the clusterProfiler package (version 3.12.0; up to September 2017) [11]. KEGG helps to better understand the advanced functions and utilities of biological systems, such as cells, organisms and ecosystems [12]. GO terms contain BP, molecular function (MF), and cellular component (CC). A *P* value < 0.05 after correction was set as the cutoff criterion.

### Univariate and multivariate Cox regression analysis

Kaplan–Meier curves were drawn, and statistical assessment was performed using the log rank test. The hazard ratio (HR) and 95% confidence interval (CI) of the association between the differentially expressed miRNA and overall survival were assessed by univariate Cox regression analysis [13]. The differentially expressed miRNAs with *P* < 0.01 were identified to be related with prognosis; multivariate Cox regression analysis was then performed. According to the cutoff of expression value, we made survival analysis. Furthermore, an Akaike information criterion (AIC)‐based stepwise factor reduction was performed to evaluate the goodness of fit of the model.

### The ceRNA network construction

After identification of differentially expressed miRNAs related with prognosis, the ceRNA network was built. The miRNA–mRNA pairing relationship was extracted by miRDB (http://www.mirdb.org) and miRTarBase (http://mirtarbase.mbc.nctu.edu.tw) databases. The targeted mRNAs were identified based on seven differentially expressed miRNAs related with prognosis and differentially expressed mRNAs in the blue coexpression module. Next, the miRNA–lncRNA pairing relationship was extracted from the miRcode (http://www.mircode.org) database, and the targeted lncRNAs were identified based on the seven miRNAs and the differentially expressed lncRNAs in the blue coexpression module. Finally, the ceRNA network was visualized using cytoscape (version 3.12.0).

### RNA extraction and RT‐qPCR

According to the manufacturer's instructions, total RNA was extracted from 22 pairs of hepatocellular carcinoma tissues and adjacent normal tissues using TRIzol. To detect miRNA expression, we synthesized cDNA with the miScript Reverse Transcription Kit (Qiagen, Hilden, Germany). Afterward, qPCR was carried out with the miScript SYBR Green PCR Kit (Qiagen). Glyceraldehyde‐3 phosphate dehydrogenase (GAPDH) was used as an internal control. The relative expression levels of miRNAs were calculated with the 2^−∆∆Ct^ method. The specific primers for miRNAs are listed in Table 2. This study was approved by the Ethics Committee of People's Hospital of Yichun City (2019002). All patients provided written informed consent. This study was strictly in line with the standards set by the Declaration of Helsinki.

**Table 2:** Primer information for RT‐qPCR.

miRNAs	Primer sequences (5′–3′)
miR‐105‐5p	5′‐TCGGCAGGTCAAATGCTCAGACTCC‐3′ 5′‐CTCAACTGGTGTCGTGGA‐3′
miR‐139‐5p	5′‐CTCGAGATTTTTGTATTATTAACTGT‐3′ 5′‐CTCAACTGGTGTCGTGGA‐3′
miR‐500a‐3p	5′‐TTGAACCAAGGTTCGTAAATACCAA‐3′ 5′‐CTCAACTGGTGTCGTGGA‐3′
miR‐767‐5p	5′‐CTCAACTGGTGTCGTGGAGTCGGCAA‐3′ 5′‐CTCAACTGGTGTCGTGGA‐3′
miR‐1180‐3p	5′‐TCGGCAGGTTTCCGGCTCGCGTGG‐3′ 5′‐CTCAACTGGTGTCGTGGA‐3′
miR‐1266‐5p	5′‐GCCGAGCCTCAGGGCTGTAGAAC‐3′ 5′‐CTCAACTGGTGTCGTGGA‐3′
miR‐4746‐5p	5′‐TCGGCAGGCCGGTCCCAGGAGAAC‐3′ 5′‐CTCAACTGGTGTCGTGGA‐3′

## Results

### Identification of differentially expressed lncRNAs, miRNAs and mRNAs

**Fig. 2:**
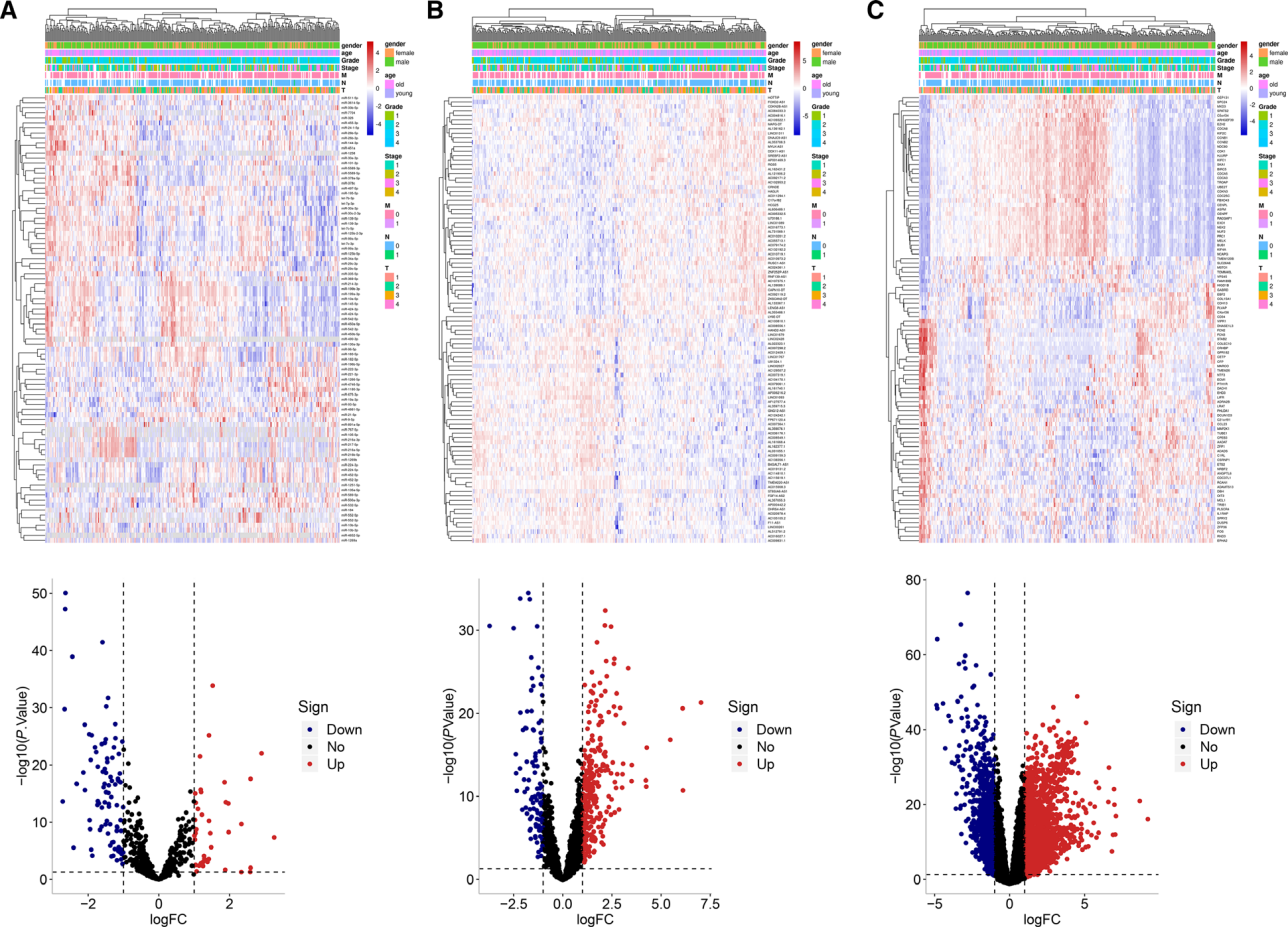
Differentially expressed lncRNAs, miRNAs and mRNAs with the cutoff of *P* < 0.05 and |logFC| > 1 in liver cancer. Volcano plots showed differentially expressed lncRNAs (including 107 down‐regulated and 298 up‐regulated lncRNAs), miRNAs (including 90 down‐regulated and 39 up‐regulated miRNAs) and mRNAs (910 down‐regulated and 1878 up‐regulated mRNAs). Heatmap showed the top 50 differentially expressed lncRNAs, miRNAs and mRNAs according to *P* value. (A) miRNAs; (B) lncRNAs; (C) mRNAs. Tumor samples were divided according to sex, age, grade, stage and TNM.

A total of 129 differentially expressed miRNAs (including 90 down‐regulated and 39 up‐regulated miRNAs) were screened according to adjusted *P* < 0.05 and |logFC| > 1 using edgeR package (Table S1 and Fig. 2A). Furthermore, differentially expressed lncRNAs and mRNAs were identified between 371 liver cancer samples and 50 adjacent normal tissue samples according to adjusted *P* < 0.05 and |logFC| > 1 using edgeR package. The results showed that there were 405 differentially expressed lncRNAs, including 107 down‐regulated and 298 up‐regulated lncRNAs (Table S2 and Fig. 2B). Furthermore, 2788 differentially expressed mRNAs, including 910 down‐regulated and 1878 up‐regulated mRNAs, were identified (Table S3 and Fig. 2C).

### Gene coexpression network construction

**Fig. 3:**
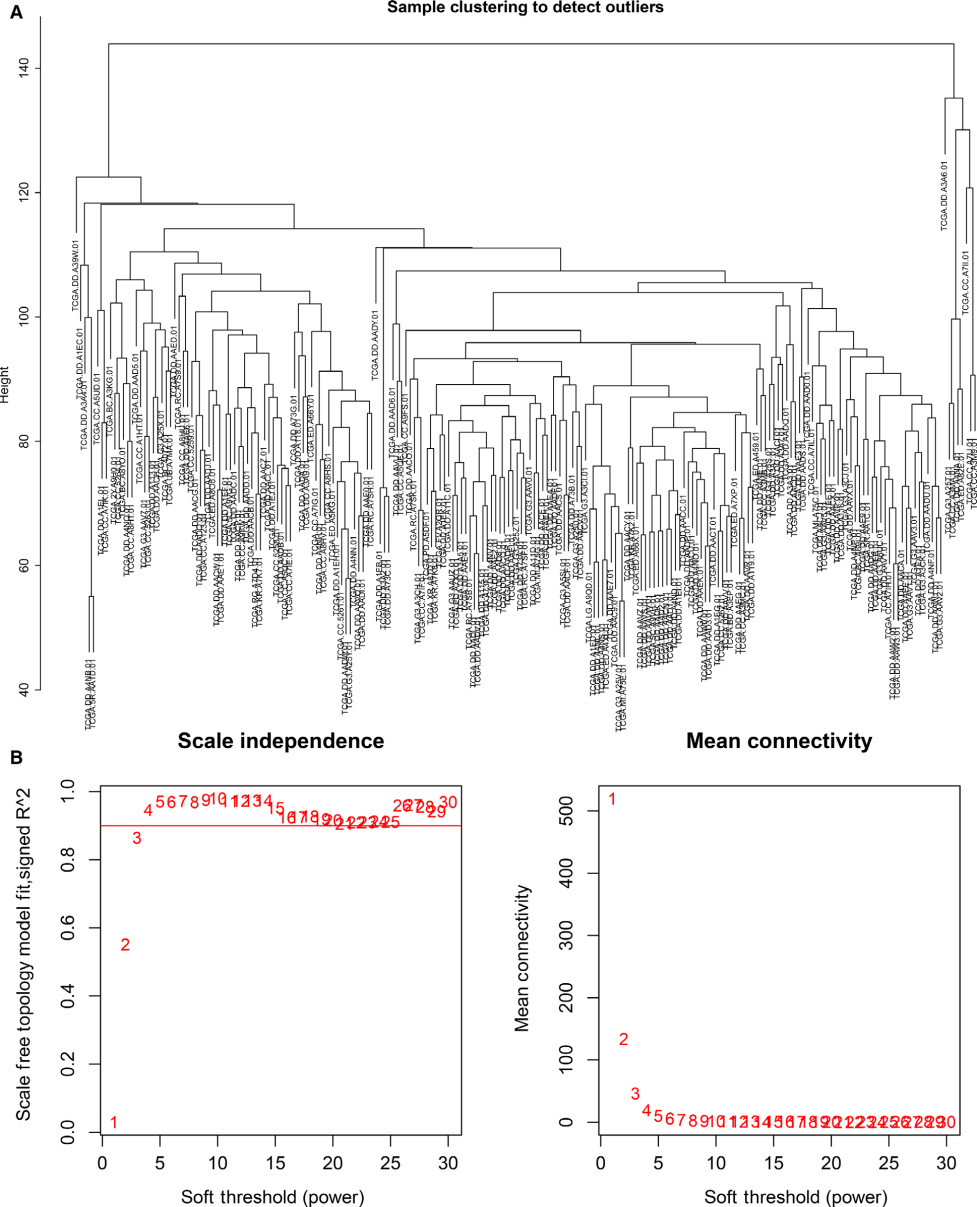
Sample clustering to detect outliers and analysis of network topology for different soft threshold powers. (A) Cluster analysis of liver cancer samples. A total of 291 liver cancer samples were clustered via the hclust tools package. (B) Analysis of network topology for different soft threshold powers on the scale independence and the mean connectivity degree.

**Fig. 4:**
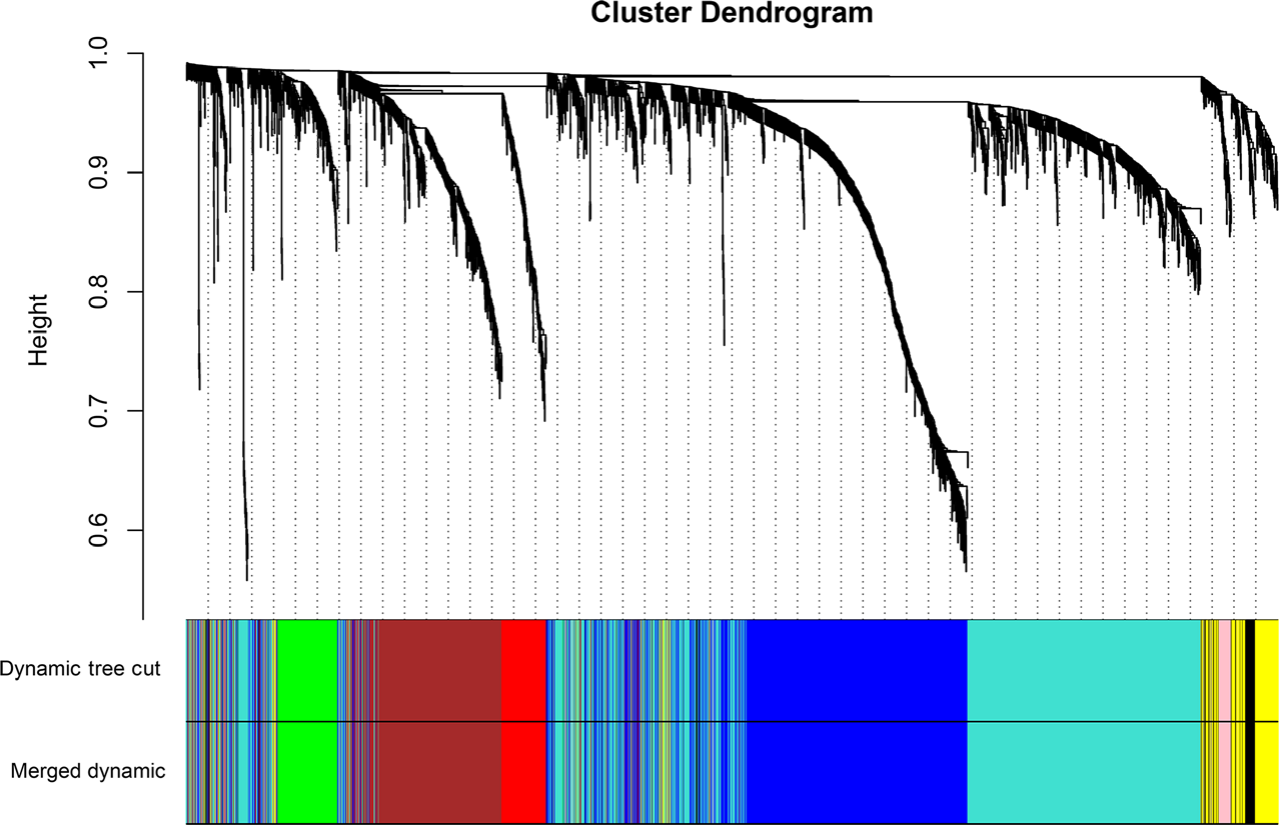
Clustering tree diagram by hierarchical clustering based on gene‐based dissimilarity measures. Different modules are identified by the dynamic tree cutting method. According to the relevance of the module, nine modules are generated after the merge. Under the tree, each color (turquoise, brown, red, green, blue, pink, black, yellow and gray) represents a module (MEturquoise, MEbrown, MEred, MEgreen, MEblue, MEpink, MEblack, MEyellow and MEgray, respectively).

**Fig. 5:**
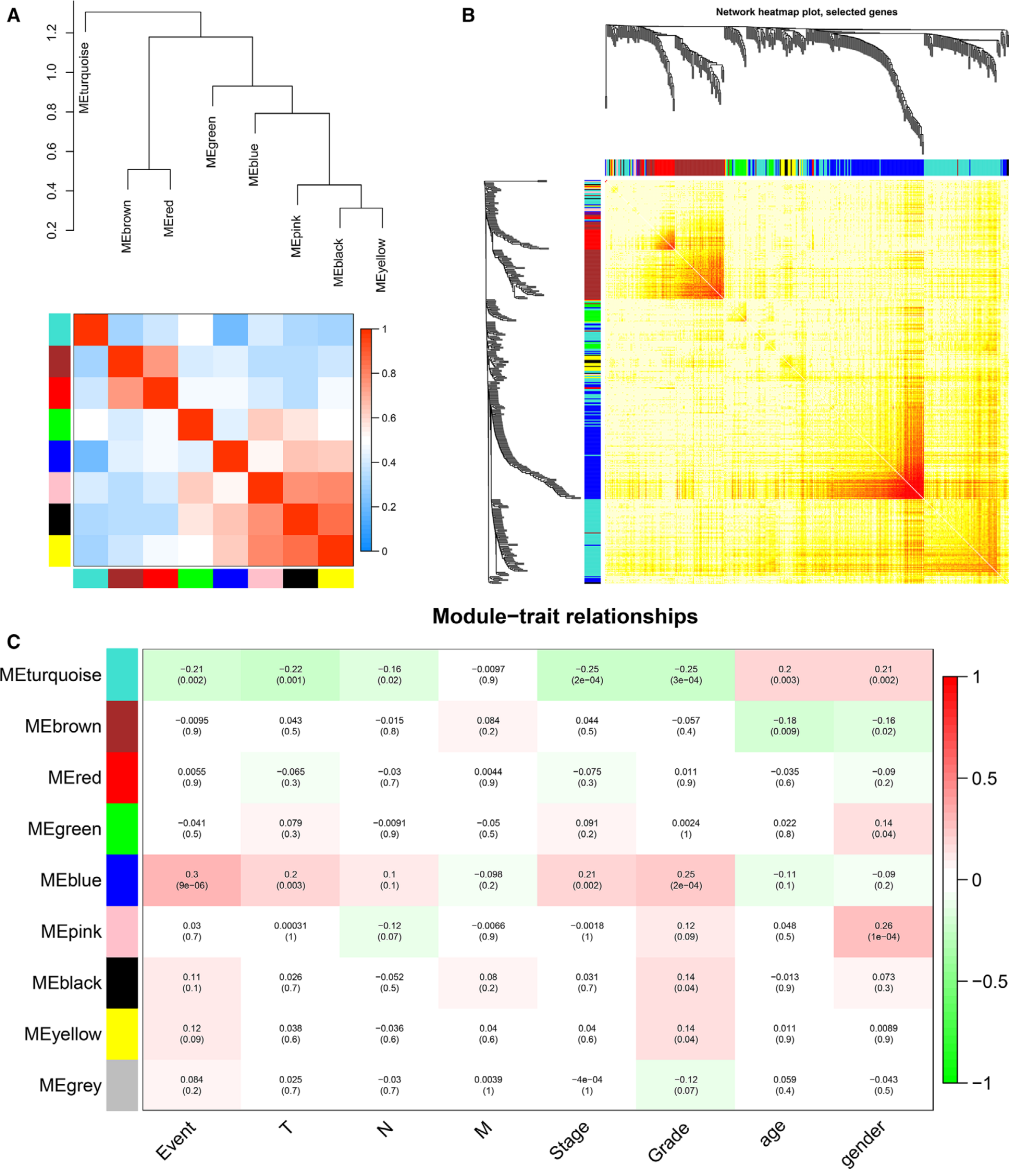
The construction of nine coexpression modules. (A) The correlation between the module and the clinical features by the eigengene network, including the dendrogram and heatmap. Red suggests a positive correlation of high adjacency, while blue indicates a negative correlation with low adjacency. (B) Network heatmap. Light colors (yellow) stand for low overlap, and red suggests high overlap. (C) Module–trait relationship construction. Each row corresponds to one ME, and each column corresponds to one feature. Each cell includes the corresponding correlation and *P* value. Pearson's correlation test is used. The table is color coded by correlation based on the color legend.

The outlier samples whose connectivity was less than −2.5 were excluded. Finally, the cluster analysis of 291 liver cancer samples was performed via the hclust tools package (Fig. 3A). After removing the outlier samples, the power value was calculated (Fig. 3B). The coexpression module was built by hierarchical clustering and dynamic branch cutting (Fig. 4). To explore the interaction between these coexpression modules, we calculated the connectivity of MEs and performed clustering analysis. Of these modules, nine coexpression modules with similar MEs were merged (Fig. 4). The gray module stands for the gene set that is not assigned to any module. The eigengene dendrogram and heatmap were performed to identify modules of correlated eigengenes, and the dendrogram suggested that these modules were associated with liver cancer clinical features (Fig. 5A,B).

### Module–trait relationship construction

**Fig. 6:**
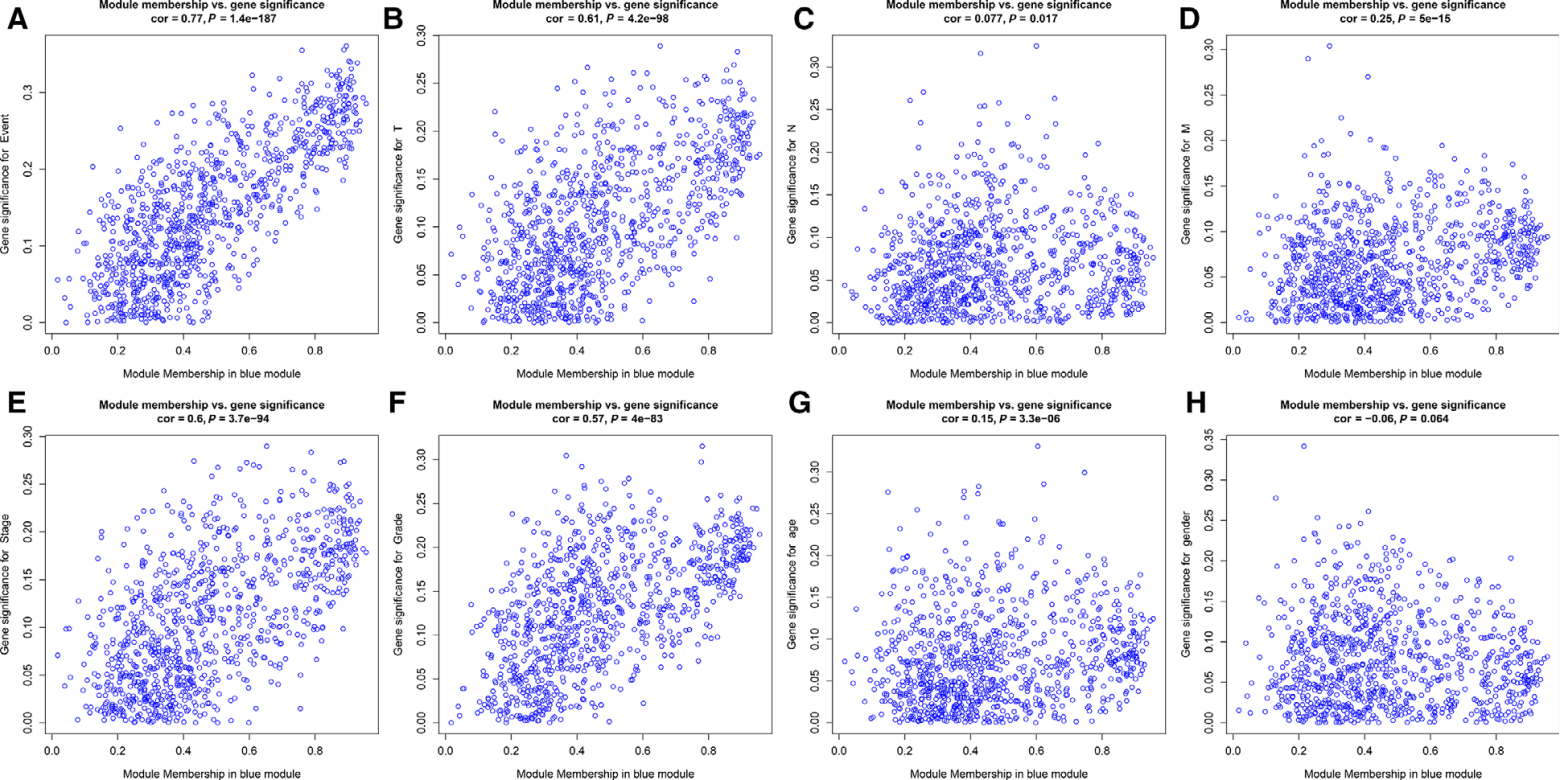
(A–H) Scatterplots of MM versus Gene Significance in the blue module.

The relationships between nine coexpression modules and clinical traits are shown in Fig. 5C. Among them, the seven modules (turquoise, brown, green, blue, pink, black and yellow) had significant associations with liver cancer clinical traits, including event, TNM, stage, grade, age and gender. We found that blue module was associated with event (*r* = 0.3, *P* = 9e−6), T (*r* = 0.2, *P* = 0.003), stage (*r* = 0.21, *P* = 0.002) and grade (*r* = 0.25, *P* = 2e−4). Therefore, we further analyzed the genes in the blue module. A scatterplot of Gene Significance versus MM in the blue module is shown in Fig. 6A–H. There is a highly significant correlation between gene significance (for event, TNM, stage, grade and age) and MM in the blue module. The genes in the blue module are listed in Table S4.

### Function enrichment analysis of genes in interested module

**Fig. 7:**
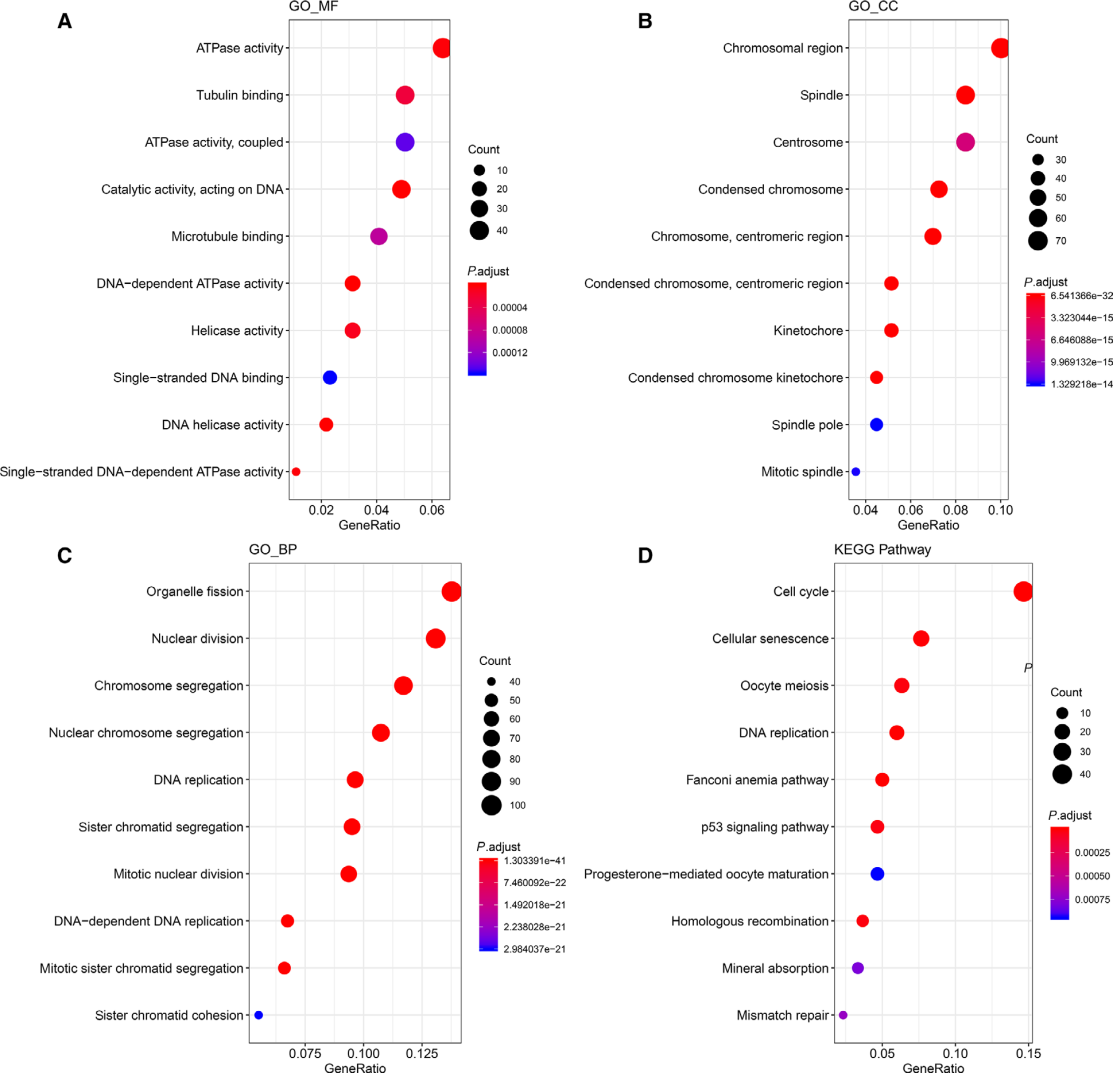
Function enrichment analysis of differentially expressed genes in the blue module. (A–C) The top 10 GO terms, including MF, CC and BP. (D) The top 10 KEGG pathways.

To further explore the function of differentially expressed genes in blue module, we conducted function enrichment analysis, including GO and KEGG analysis. The top 10 GO terms, including MF, CC and BP, were shown in Fig. 7A–C. We found that the differentially expressed genes were mainly enriched in several pathways, such as ATPase activity, chromosomal region, organelle fission and so on. Also, these differentially expressed genes were mainly enriched in KEGG pathways, such as cell cycle, cellular senescence, oocyte meiosis, DNA replication, Fanconi anemia pathway, p53 signaling pathway and so on (Fig. 7D). Therefore, the differentially expressed genes in blue module could participate in the occurrence and development of liver cancer.

### Survival analysis and ceRNA network construction

**Fig. 8:**
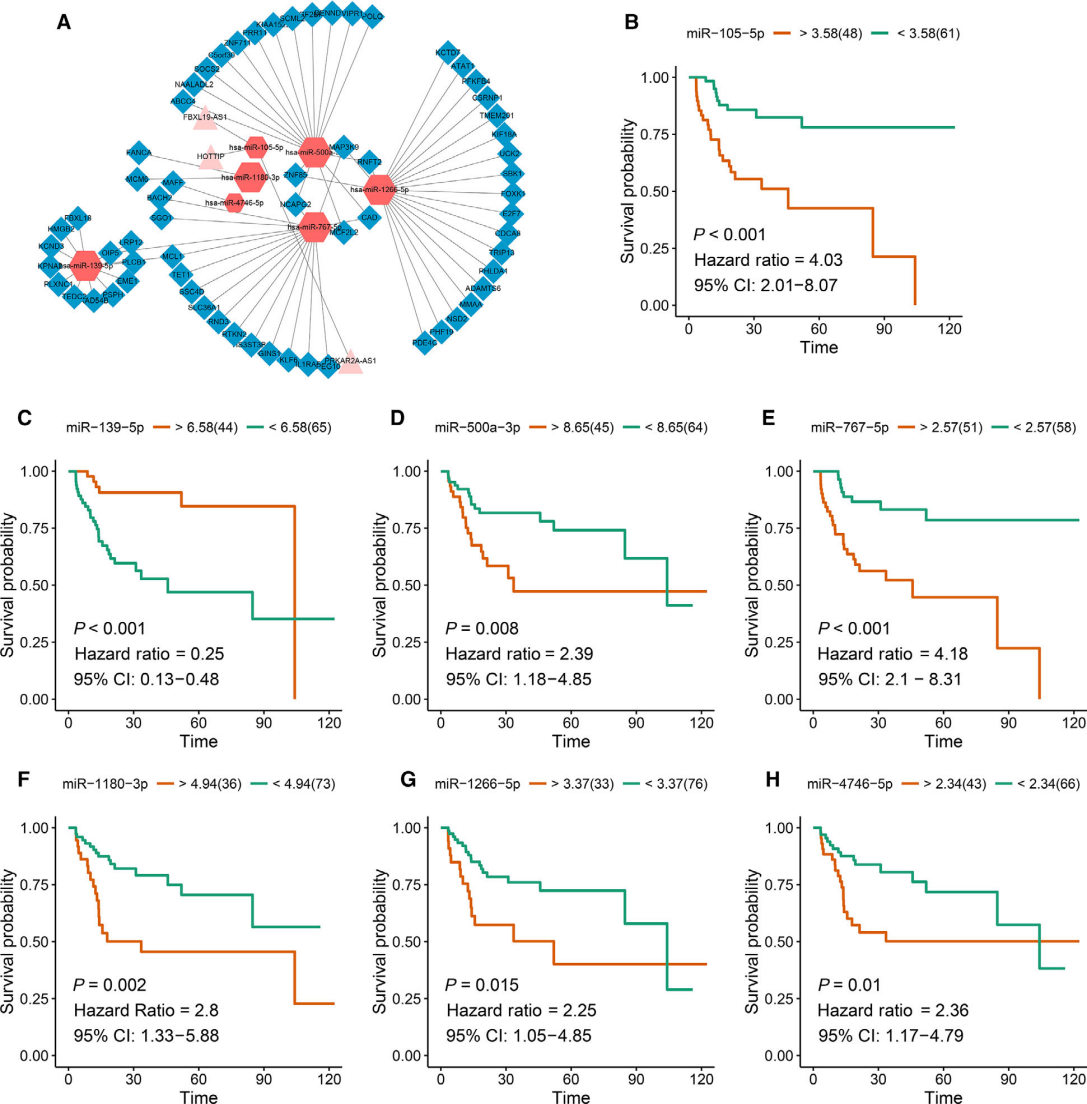
Survival analysis and ceRNA network construction. (A) The ceRNA network for liver cancer prognosis. Rhombus: mRNAs; triangle: lncRNAs; hexagon: miRNAs. (B–H) Survival analysis of seven differentially expressed miRNAs.

**Fig. 9:**
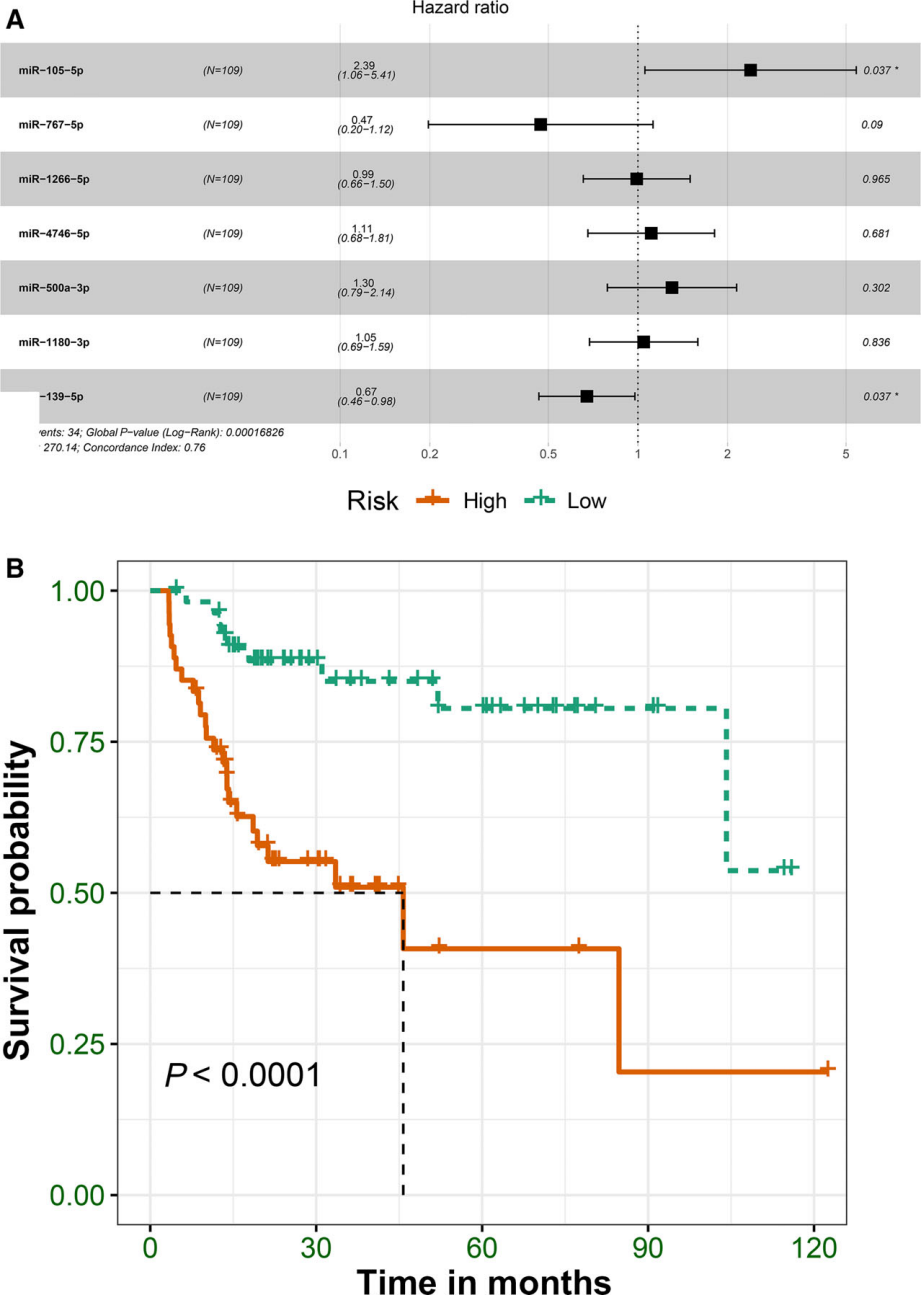
The seven‐miRNA model for liver cancer. (A) Multivariate Cox regression analysis of seven miRNAs in liver cancer. *N* = 109. HR and CI were calculated for each miRNA. (B) Survival analysis of seven‐miRNA model.

After extracting the differentially expressed genes in blue module, we performed univariate Cox regression analysis to identify differentially expressed miRNAs related with prognosis, which were used to construct the ceRNA network (*P* < 0.001). After matching miRNA–mRNA and miRNA–lncRNA relationships, the ceRNA network was built. There were 72 mRNAs, 3 lncRNAs and 7 miRNAs, all of which were closely related with prognosis (Fig. 8A). The miRNA–mRNA pairs are listed in Table S5. Univariate and multivariate analyses of various prognostic parameters in patients with liver cancer were performed. As shown in Table 3, univariate analysis results showed that T classification and M classification were closely correlated with overall survival of patients with liver cancer. Furthermore, T classification could become an independent prognostic factor for liver cancer according to multivariate analysis results. There were seven miRNAs closely related with liver cancer prognosis, including miR‐105‐5p (*P* < 0.001, HR = 4.03, 95% CI: 2.01–8.07), miR‐767‐5p (*P* < 0.001, HR = 4.18, 95% CI: 2.1–8.31), miR‐1266‐5p (*P* = 0.015, HR = 2.25, 95% CI: 1.05–4.85), miR‐4746‐5p (*P* = 0.01, HR = 2.36, 95% CI: 1.17–4.79), miR‐500a‐3p (*P* = 0.008, HR = 2.39, 95% CI: 1.18–4.85), miR‐1180‐3p (*P* = 0.002, HR = 2.8, 95% CI: 1.33–5.88) and miR‐139‐5p (*P* < 0.001, HR = 0.25, 95% CI: 0.13–0.48; Fig. 8B–H). Multivariate Cox regression analysis results revealed that miR‐105‐5p (*P* = 0.037, HR = 2.39, 95% CI: 1.06–5.41) and miR‐139‐5p (*P* = 0.037, HR = 0.67, 95% CI: 0.46–0.98) could be considered as independent factors (Fig. 9A). The seven‐miRNA model was constructed, and survival analysis demonstrated that the model could significantly distinguish prognosis differences between high‐risk and low‐risk groups (Fig. 9B). Furthermore, we further validated the efficiency of the seven‐miRNA model for prediction of liver cancer prognosis. We first calculated the score of each sample through the seven‐miRNA model. The linear regression model after integrating various factors showed that the risk value was significantly associated with prognosis (Table 4). The AIC value of the risk score + stage integrated model was the smallest through AIC, where risk score was smaller than stage, reflecting the effectiveness of the seven‐miRNA model for liver cancer prognosis. The median value of the model was used to differentiate the samples into high‐ and low‐risk groups, as shown in Table 5. As expected, there were significant differences in several clinical subtypes. Thus, the seven‐miRNA model could become a better prediction model related to liver cancer prognosis.

**Table 3:** Univariate and multivariate analyses of various prognostic parameters in patients with liver cancer.

Characteristics	Univariate analysis			Multivariate analysis		
	*P* value	HR	95% CI	*P* value	HR	95% CI
Age	0.379	1.001	0.992–1.021	0.971	1	0.981–1.02
Sex	0.516	0.879	0.595–1.298	0.413	1.27	0.715–2.26
Grade	0.270	1.154	0.895–1.488	0.231	1.25	0.869–1.79
T	<0.0001*	1.867	1.532–2.275	<0.0001*	2.02	1.53–2.66
N	0.215	2.442	0.595–10.020	0.101	3.36	0.789–14.3
M	0.01**	4.750	1.486–15.187	0.442	1.66	0.458–5.99

* *P* < 0.0001.

** *P* < 0.01.

**Table 4:** Linear regression model for liver cancer data. Residual standard error (SE): 0.4203 on 197 degrees of freedom. Multiple *R* ^2^: 0.2124, adjusted *R* ^2^: 0.1604. *F*‐statistic: 4.086 on 13 and 197 degrees of freedom, *P* value: 5.789e−6. NA, not applicable.

Characteristics	Estimate	SE	*t* value	Pr(>|*t*|)
(Intercept)	−0.09333	0.18795	−0.497	0.62005
Risk	0.07877	0.02364	3.332	0.00103*
Age	0.002719	0.002428	1.12	0.26417
Sex				
Male	−0.02298	0.065533	−0.351	0.72624
Grade				
G2	0.019481	0.100283	0.194	0.84618
G3	0.03957	0.100324	0.394	0.6937
Missing	0.193021	0.16169	1.194	0.234
Stage				
II	0.667785	0.618218	1.08	0.28138
III	−0.2558	0.635633	−0.402	0.6878
IV	0.221274	0.687636	0.322	0.74795
T				
T2	−0.59862	0.611494	−0.979	0.32881
T3	0.560992	0.631476	0.888	0.37542
T4	0.553129	0.553129	0.86	0.39109
N				
N1	0.68285	0.441278	1.547	0.12336
M				
M1	NA	NA	NA	NA

* *P* < 0.01.

**Table 5:** Clinical characteristics between high and low risks for patients with liver cancer.

Characteristics	Total (*N* = 211)	High risk (*n* = 106)	Low risk (*n* = 105)
Age, *n* (%)			
<60 years	115 (54.5%)	63 (59.43%)	52 (49.52%)
≥60 years	96 (45.5%)	43 (40.57%)	53 (50.48%)
Sex, *n* (%)			
Female	63 (29.86%)	37 (34.91%)	26 (24.76%)
Male	148 (70.14%)	69 (65.09%)	79 (75.24%)
Grade, *n* (%)			
G1	24 (11.37%)	10 (9.43%)	14 (13.33%)
G2	92 (43.6%)	38 (35.85%)	54 (51.43%)
G3	85 (40.28%)	52 (49.06%)	33 (31.43%)
Missing	10 (4.74%)	6 (5.66%)	4 (3.81%)
Stage, *n* (%)			
I	104 (49.29%)	48 (45.28%)	56 (53.33%)
II	44 (20.85%)	23 (21.7%)	21 (20%)
III	60 (28.44%)	33 (31.13%)	27 (25.71%)
IV	3 (1.42%)	2 (1.89%)	1 (0.95%)
T, *n* (%)			
T1	105 (49.76%)	49 (46.23%)	56 (53.33%)
T2	45 (21.33%)	23 (21.7%)	22 (20.95%)
T3	52 (24.64%)	28 (26.42%)	24 (22.86%)
T4	9 (4.27%)	6 (5.66%)	3 (2.86%)
N, *n* (%)			
N0	208 (98.58%)	104 (98.11%)	104 (99.05%)
N1	3 (1.42%)	2 (1.89%)	1 (0.95%)
M, *n* (%)			
M0	208 (98.58%)	104 (98.11%)	104 (99.05%)
M1	3 (1.42%)	2 (1.89%)	1 (0.95%)

### Validation of seven miRNAs using RT‐qPCR

**Fig. 10:**
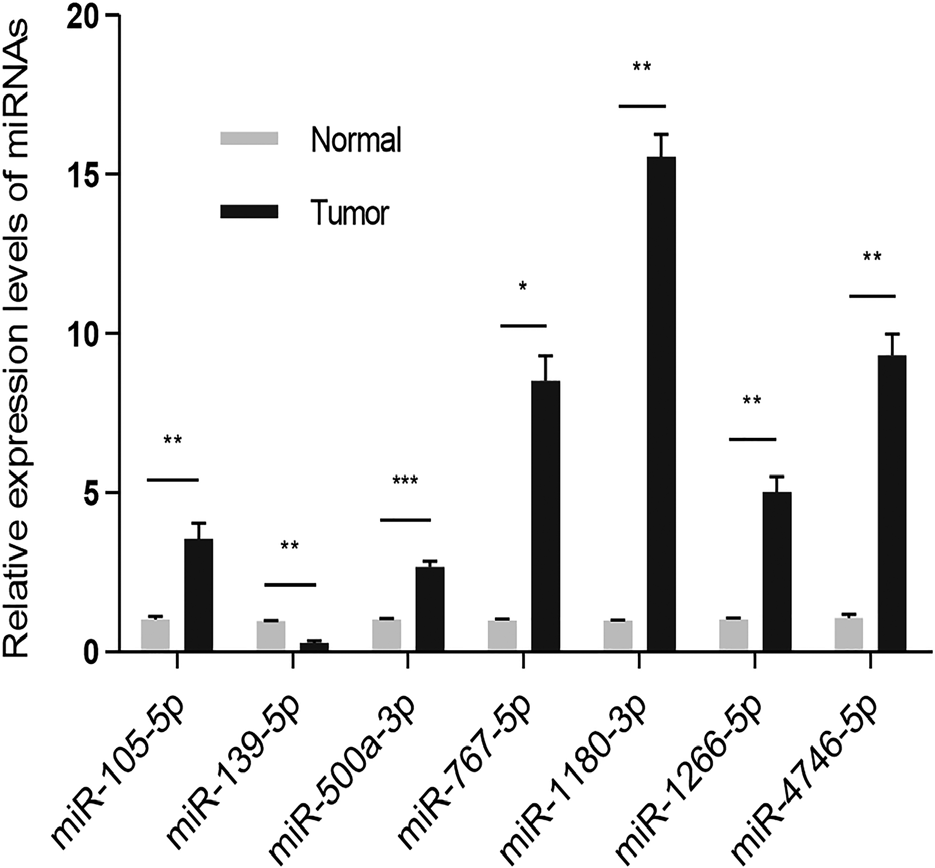
Validation of seven miRNAs using RT‐qPCR. *N* = 22. The data were expressed as the mean ± standard error of mean. *P < 0.05; **P < 0.01; ***P < 0.001.

To further validate the seven miRNAs in liver cancer, we performed RT‐qPCR based on 22 pairs of hepatocellular carcinoma tissues and adjacent normal tissues. Consistent with our bioinformatics analysis results, RT‐qPCR results showed that the expression levels of miR‐105‐5p (*P* < 0.01), miR‐500a‐3p (*P* < 0.001), miR‐767‐5p (*P* < 0.05), miR‐1180‐3p (*P* < 0.01), miR‐1266‐5p (*P* < 0.01) and miR‐4746‐5p (*P* < 0.01) were higher in liver cancer tissues than those in adjacent normal tissues. Furthermore, the expression levels of miR‐139‐5p (*P* < 0.01) were lower in liver cancer tissues than that in adjacent normal tissues (Fig. 10).

## Discussion

As molecular biomarkers continue to develop, prognostic indicators will be promising clinical tools for liver cancer [14]. WGCNA has been widely applied to screen novel markers [15]. In the coexpression networks, genes with similar expression patterns are clustered together in the same module, and these genes may have similar regulatory functions [16]. In addition, to explore the genetic mechanisms behind clinical features, the relationship between modules and clinical features was identified. The link between clinical features and genes in the module may help to understand the pathogenesis of liver cancer and screen for potential biomarkers. The results showed that the blue module is significantly related to event, T, stage and grade. Function enrichment analysis demonstrated that the differentially expressed genes were mainly enriched in several KEGG pathways, such as cell cycle, cellular senescence, oocyte meiosis, DNA replication, Fanconi anemia pathway, p53 signaling pathway and so on. Therefore, the differentially expressed genes in the blue module could participate in the occurrence and development of liver cancer.

Therefore, we further analyzed the genes in this module. To further clarify biomarkers that can serve as prognostic factors, we used univariate Cox regression analysis to identify miRNAs associated with prognosis. The results revealed that seven differentially expressed miRNAs were closely related with prognosis. After matching miRNA–mRNA and miRNA–lncRNA relationships, the ceRNA network was then constructed and the seven miRNAs were identified in the network. The hub miRNAs represent the primary regulatory role of the blue module. To further confirm the prognostic value of these seven miRNAs in liver cancer, we presented multivariate Cox regression analysis. The results showed that the seven‐miRNA model has a high sensitivity in predicting the prognosis of patients with liver cancer, and miR‐105‐5p and miR‐139‐5p could be an independent prognostic factor compared with other miRNAs. Survival analysis revealed that the seven‐miRNA module had significant correlation with prognosis of liver cancer. More importantly, RT‐qPCR confirmed that miR‐105‐5p, miR‐500a‐3p, miR‐767‐5p, miR‐1180‐3p, miR‐1266‐5p and miR‐4746‐5p were up‐regulated in liver cancer tissues compared with normal tissues. Furthermore, miR‐139‐5p was down‐regulated in liver cancer tissues compared with normal tissues.

Human miR‐105 is located in the intron region of GABR^A3A^, which is located on the X chromosome [17]. Compared with normal tissues, miR‐105 is down‐regulated in many malignant tumors as a tumor suppressor or oncogene, such as breast cancer, non‐small cell lung cancer, gliomas, colorectal cancer and so on [18, 19, 20, 21]. Increasing evidence suggests that miR‐105 can be used as a prognostic predictor, because its expression pattern is closely associated with prognosis of these cancers. It has been confirmed that miR‐105 is down‐regulated in hepatocellular cancer cell lines and tissues, which promotes proliferation and tumorigenicity of hepatocellular cancer cells *in vitro* and *in vivo* [22]. Furthermore, miR‐105 acts as a tumor suppressor in hepatocellular carcinoma via inhibiting the phosphatidylinositol 3‐kinase (PI3K)/AKT signaling pathway. In our study, univariate and multivariate Cox regression analysis showed that miR‐105‐5p could become an independent prognostic factor for liver cancer. miR‐767 is up‐regulated in human melanoma tissues and cell lines, which promote melanoma cell proliferation, and miR‐767 acts as a tumor promoter in human melanoma by targeting CYLD [23]. In addition, miR‐767 could become a prognostic factor for thyroid cancer [24]. It has been confirmed that miR‐1266 contributes to several cancers. For example, miR‐1266‐5p is down‐regulated in prostate cancer, which regulates the apoptotic pathway by targeting the antiapoptotic genes *BCL2* and *BCL2L1* [25]. Human telomerase reverse transcriptase (hTERT) is a catalytic subunit of the telomerase complex, and its increased expression is associated with the expansion and metastasis of gastric cancer. miR‐1266 is identified as a hTERT inhibitor in gastric cancer, which interacts with the 3′ UTR of hTERT, whereas miR‐1266 is significantly reduced in gastric cancer tissues [26]. miR‐1266 is significantly elevated in pancreatic cancer, which is associated with poor survival and chemotherapy response in patients with pancreatic cancer [27]. miR‐4746 is differentially expressed in several cancers through pan‐cancer analysis [28]. miR‐500a‐3p is down‐regulated in lung cancer, which is associated with poor prognosis in patients with lung cancer [29]. In addition, miRNA‐500a‐3p suppresses cell proliferation and invasion in human non‐small cell lung cancer [30]. miR‐500a‐3p is highly expressed in hepatocellular carcinoma tissues and cells, which is associated with the prognosis of patients with hepatocellular carcinoma. Moreover, miR‐500a‐3p promotes cancer stem cell characteristics by activating the JAK/STAT3 signaling pathway [31]. The expression of miR‐1180 is significantly increased in hepatocellular carcinoma cells and tissues, which promotes cell proliferation of hepatocellular carcinoma by targeting TNFAIP3 interacting protein 2 (TNIP2) [32]. Apoptosis resistance in human hepatocellular carcinoma is an important factor in carcinogenesis. The ectopic expression of miR‐1180 has an antiapoptotic effect in hepatocellular carcinoma [33]. miR‐139‐5p plays a role in aerobic glycolysis, cell proliferation, migration, invasion and metastasis [34, 35]. In addition, miR‐139‐5p is significantly associated with recurrence of hepatocellular carcinoma [36]. The earlier analysis revealed that the seven miRNAs could contribute to the development of liver cancer. Abnormal expression of these miRNAs could predict prognosis of liver cancer. Several similar studies have the similar aims and objectives to identify the miRNA using Gene Expression Omnibus and TCGA databases. Wang *et al*. [37] identified several miRNAs that could become potential prognostic biomarkers for liver cancer by bioinformatics analysis. Li *et al*. [21] identified novel prognostic biomarkers for liver cancer by constructing a coexpression network. Furthermore, Zhang *et al*. [38] reported that lncRNAs had differential expression patterns and ceRNA potential in liver cancer between 372 liver cancer tissues and 48 adjacent normal tissues from TCGA and Gene Expression Omnibus databases. However, these biomarkers were not validated by basic experiments. In our study, we first constructed nine coexpression modules by WGCNA, and the blue module had a significant association with clinical traits of liver cancer. Furthermore, the genes in the blue module could participate in many signaling pathways. After identifying seven miRNAs related with prognosis, the ceRNA network revealed that the seven miRNAs had a complex regulatory network. Moreover, the seven‐miRNA module could predict prognosis of patients with liver cancer. In our study, RT‐qPCR results confirmed the expression patterns of seven miRNAs in liver cancer tissues compared with adjacent normal tissues. Therefore, the function of the seven miRNAs in liver cancer are worth more in‐depth research.

## Conclusion

In our study, we constructed gene coexpression modules related with clinical traits of liver cancer. Seven miRNAs were identified as prognostic biomarkers by univariate and multivariate Cox regression analysis. Furthermore, the seven‐miRNA module possesses potential value to predict prognosis of liver cancer. Therefore, our study constructed coexpression modules by WGCNA and identified prognostic biomarkers for liver cancer.

## Conflict of interest

The authors declare no conflict of interest.

## Author contributions

YZ conceived and designed the study. BS conducted most of the experiments and data analysis, and wrote the manuscript. KL participated in collecting data and helped to draft the manuscript. All authors reviewed and approved the manuscript.

## Supporting information


**Table S1.** Differentially expressed miRNAs for liver cancer.

**Table S2.** Differentially expressed lncRNAs for liver cancer.

**Table S3.** Differentially expressed mRNAs for liver cancer.

**Table S4.** The genes in the blue module.

**Table S5.** The miRNA–mRNA pairs in the ceRNA network.

## Data Availability

The datasets used and/or analyzed during this study are available from the corresponding author on reasonable request.
